# Epidemiology Characteristics of COVID-19 Infection Amongst Primary Health Care Workers in Qatar: March-October 2020

**DOI:** 10.3389/fpubh.2021.679254

**Published:** 2021-05-20

**Authors:** Mohamed Ghaith Al-Kuwari, Mariam Ali AbdulMalik, Asma Ali Al-Nuaimi, Jazeel Abdulmajeed, Hamad Eid Al-Romaihi, Sandy Semaan, Mujeeb Kandy

**Affiliations:** ^1^Primary Health Care Corporation, Doha, Qatar; ^2^Ministry of Public Health, Doha, Qatar

**Keywords:** occupational health, healthcare workers, COVID-19, occupational disease, infectious disease, occupational exposure, primary care

## Abstract

**Background:** COVID-19 transmission was significant among Healthcare workers worldwide. In March 2020, Qatar started reporting numbers of COVID-19 positive cases among workers in Primary Health Care Corporation (PHCC). The study estimates the burden of the aforementioned infections and examines the demographic characteristics associated with the recorded positivity rates.

**Method:** A cross-sectional descriptive study was conducted among Primary healthcare workers between March 1st and October 31st, 2020. The study examined the positivity rate of the different types of Primary healthcare workers and, analyzed the demographic characteristics of the infected persons.

**Results:** 1,048 (87.4%) of the infected Health Care Workers (HCWs) belonged to the age group below 45 years, and 488 (40.7%) HCWs were females. 450 (37.5%) were HCWs clinical staff working in one of the 27 PHCC Health Centers (HCs) Despite the increased patient footfall and risk environment, the COVID dedicated HCs had an attack rate of 10.1%, which is not significantly different from the average attack rate of 8.9% among staff located in other HCs (*p* = 0.26). Storekeepers, engineering & maintenance staff, housekeeping staff, support staff, and security staff (outsourced non-clinical positions) had the highest positivity rates, 100, 67.2, 47.1, 32.4, and 29.5% respectively.

**Conclusion:** The elevated risk of infection among outsourced non-clinical healthcare workers can be explained by environmental factors such as living conditions. Furthermore, better containment within clinical healthcare workers can be attributed to strict safety training and compliance with preventative measures which is recommended to be implemented across all settings.

## Introduction

COVID-19 disease has affected more than 100 million individuals worldwide. Health care workers (HCWs) are at increased risk of contracting infectious diseases because of their occupational exposure (1). In the State of Qatar, more than 150,000 people were infected resulting in ~200 deaths (2). This has taken many public health measures such as social distancing strategies to protect its population from COVID-19 disease and to reduce the incidence of new cases, as no specific pharmaceutical intervention was available during the first surge of the pandemic in 2020 (3).

As part of the State of Qatar's efforts to control the COVID-19 pandemic, Primary Healthcare Corporation (PHCC) has had a frontline presence and a proactive role in reducing the spread of coronavirus in Qatar, with dedicated COVID-19 Center, contact tracing, and dedicated drive through swabbing hubs to assist with early detection (4).

The Corporation comprises a network of 27 health centers and employs more than 6,000 employees.

International studies have also estimated that frontline healthcare workers had a higher risk of reporting a positive test than people living in the general community, adjusting for the likelihood of receiving a test (5, 6), and the prevalence of exposed workers in the healthcare industry (7, 8).

While adult patients usually present typical symptoms like fever, cough, taste, and smell disorders, pediatric clinical signs are less severe, making the diagnosis challenging to interpret and increasing the risk of contagion for healthcare workers (9).

A national study in Qatar has identified that COVID-19 infection often occurs with HCWs who are not directly working with COVID-19 patients. One of the reasons depicted is that Personal Protective Equipment (PPE) use is less stringent in such settings (10).

However, there is still limited information available about COVID-19 epidemiological characteristics among HCWs, and it varies in different geographical regions of the world (11, 12). Understanding the epidemiology of COVID-19 infection among healthcare workers at primary care settings is a crucial factor in determining the outbreak trajectories and clinical outcomes at the population level, considering their extent of interaction with the health seeking population in times of a health emergency.

In this study, we aim to estimate the burden of COVID-19 infection amongst all types of workers active at PHCC and identify specific health care workgroups who may be particularly vulnerable to the disease during the ongoing COVID-19 pandemic.

## Materials and Methods

### Method

A cross-sectional descriptive study was conducted to study the burden of COVID-19 among HCWs working at PHCC during the COVID-19 pandemic and analyze the demographic characteristics of the infected HCWs. All HCWs who tested positive for COVID-19 during the period from March 1st to October 31st, 2020, were included for analysis.

### Definitions

For this study, a healthcare worker is defined as any person serving in a PHCC healthcare setting, either directly hired or a contractual employee, who had the potential for direct or indirect exposure to patients or their infectious secretions and materials, including, but not limited to, physicians, nurses, paramedics, laboratory workers, and clinical support staff, e.g., wellness gym instructors, administrative staff, facility officers, security officers, or maintenance workers.

### Material and Data Source

Secondary data available from PHCC databases were compiled and utilized for this study. Data was extracted from the PHCC staff database, including demographics of the personnel, work location during the pandemic and other related information. Subsequently, this data was mapped to the COVID-19 polymerase chain reaction (PCR) results available on Cerner electronic medical record, the Clinical Information System used by the PHCC.

The compiled data extract was imported into STATA v 15.1—(StataCorp. 2017. College Station, TX: StataCorp LLC.). Chi–square test was used as appropriate; a *p* < 0.05 was considered significant.

The attack rate (AR) was calculated as the percentage of the cumulative number of laboratory confirmed COVID-19 positive HCWs divided by the total number of HCWs. The test positivity rate (PR) was defined as the percentage of the cumulative number of laboratory-confirmed COVID-19 positive HCWs divided by the total number of HCWs tested

## Results

During the study period extending from March 1st to October 31st, 2020 PHCC employed 9,172 staff. Among the 7,407 (81%) staff who were subjected to COVID-19 RT-PCR tests, 1,199 (16.2%) were found positive. An overall attack rate of 13.1% was estimated.

**Figure 1:**
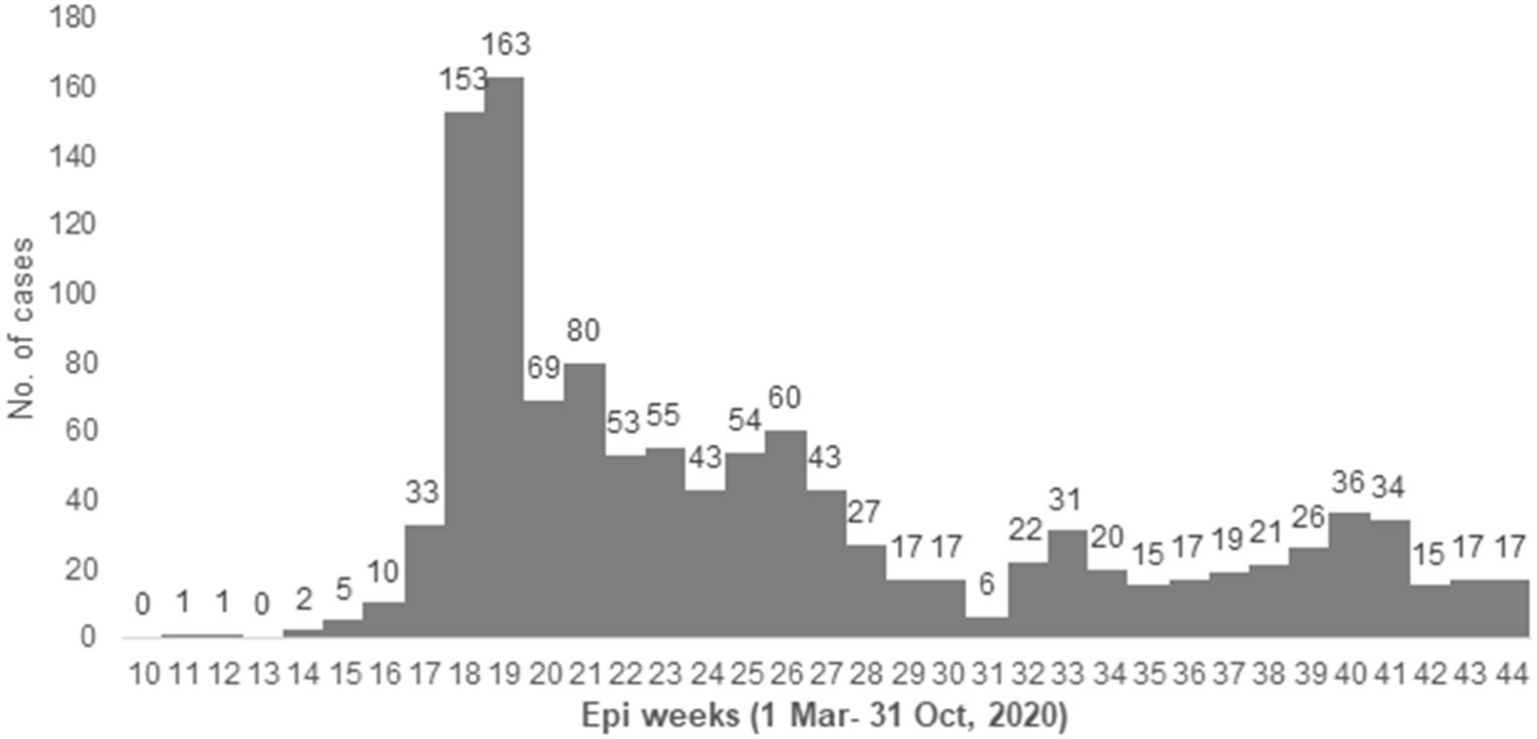
Epidemic curve with case number of HCWs with COVID-19 in PHCC from 1 March to 31 October 2020 (Epiweeks 10–44).

The first case among PHCC staff was detected on March 12th, 2020 (week 12). A major peak of cases was observed during April and May (week 18–19), as shown in Figure 1.

The median age of the infected HCWs was 36 years. 1,048 (87.4%) belonged to the age group below 45 years and 488 (40.7%) HCWs were females. 695 (58%) were directly hired regular employees of PHCC, while 450 (37.5%) HCWs were clinical staff working in one of the 27 PHCC HCs; amongst them 131 (10.9% of the infected HCWs) worked in the 4 designated COVID-19 health centers.

Significant difference was observed in the positivity rates while comparing the infected HCWs based on various variables. HCWs aged <45 years had a higher attack rate (14.5%) and test positivity (17.5%) compared to their colleagues aged above 45 years (*p* < 0.001). Male employees had a higher attack rate (18.5%) and test positivity (23.8%) compared to female employees (*p* < 0.001). Non-clinical occupations had a higher attack rate (19.7%) and test positivity (26.8%) compared to clinical occupations (*p* < 0.001). Contractual employees had a higher attack rate (42.9%) and test positivity (44.4%) compared to regular PHCC employees (*p* < 0.001).

No significant difference was observed in the infection rates of employees who worked in COVID HCs compared to those working in other PHCC HCs (*p* = 0.61). Detailed comparison of estimated rates is provided in Table 1.

**Table 1:** PHCC Staff Characteristics, screening proportion, attack rate and positivity rate (1 March−31 October).

**Variable**	**Total staff**	**Tested**	**Positives**	**Attack rate**	**Test positivity**	***p-value***
**All staff**	9,172	7,407	1,199	13.1%	16.2%	
**Age group**						*<0.001*
<45 years	7,250	5,999	1,048 (87.4%)	14.5%	17.5%	
45 years and above	1,922	1,408	151 (12.6%)	7.9%	10.7%	
**Gender**						*<0.001*
Female	5,320	4,415	488 (40.7%)	9.2%	11.1%	
Male	3,852	2,992	711 (59.3%)	18.5%	23.8%	
**Occupation**						*<0.001*
Clinical	5,363	4,610	450 (37.5%)	8.4%	9.8%	
Non-clinical	3,809	2,797	749 (62.5%)	19.7%	26.8%	
**Type of employment**						*<0.001*
Direct hire	7,996	6,271	695 (58%)	8.7%	11.1%	
Contractual	1,176	1,136	504 (42%)	42.9%	44.4%	
**Location of work**						*0.61*
Covid-19 HC	1,301	1,150	131 (10.9%)	10.1%	11.4%	
Other HC	6,001	4,893	532 (44.4%)	8.9%	10.9%	

*The italic value is the significance as explained in the methodology a p < 0.05 was considered significant*.

Among the clinical staff, all occupations have been affected by the spread of the COVID-19 with positivity rates ranging between ~6 and 12%. In particular, pharmacists, dentists, wellness gym staff, and nurses had higher positivity rates compared to the others- 12.7, 11.2, 10.7, and 10.5% respectively (Table 2).

**Table 2:** Attack rate and test positivity among clinical staff.

**Occupation**	**Total staff**	**Total tested**	**Positives**	**Attack rate (%)**	**Test positivity (%)**
Nurse	2,506	2,255	236	9.4	10.5
Physician	1,079	858	72	6.7	8.4
Pharmacist	479	403	51	10.6	12.7
Lab technician	413	350	28	6.8	8.0
Dentist	242	196	22	9.1	11.2
Dental staff	231	192	14	6.1	7.3
Radiology staff	211	189	12	5.7	6.3
Wellness gym staff	94	75	8	8.5	10.7
Physiotherapist	54	47	3	5.6	6.4
Allied health staff	48	41	4	8.3	9.8

Amongst the non-clinical occupations, storekeepers, engineering & maintenance staff, housekeeping staff, support staff, and security staff had the highest positivity rates, 100, 67.2, 47.1, 32.4, and 29.5% respectively (Table 3). Administrative Staff who are predominant amongst non-clinical staff had a positivity rate of 3.5% and an attack rate of 1.8%.

**Table 3:** Attack rate and test positivity among non-clinical staff.

	**Total staff**	**Total tested**	**Positives**	**Attack rate (%)**	**Test positivity (%)**
Administrative staff	1,326	689	24	1.8	3.5
Receptionists and cashiers	807	657	112	13.9	17.0
Housekeeping staff	530	526	248	46.8	47.1
Support staff	390	358	116	29.7	32.4
Security officers	388	322	95	24.5	29.5
Transport staff	135	82	18	13.3	22.0
Customer service staff	106	81	10	9.4	12.3
Engineering & maintenance staff	112	67	45	40.2	67.2
Storekeepers	15	15	15	100	100

Out of the 27 PHCC HCs, four HCs had been designated official assessment and triage COVID-19 HCs starting March 15, 2020 (Table 4). Similar patient volumes were seen at both these categories of HCs- 143,154 suspected patients were swabbed at the 4 COVID HCs and a cumulative number of 145,565 suspected patients were swabbed in all other HCs.

**Table 4:** Attack rate among staff in various health centers.

**PHCC facility**	**Total staff**	**Positive**	**Attack rate (%)**
**Specialized COVID health centers**	**1,301**	**131**	**10.1**
Gharrafat Al Rayyan	290	30	10.3
Muaither	327	36	11.0
Rawdat Al Khail	389	36	9.3
Umm Slal	295	29	9.8
**Other PHCC health centers**	**6,001**	**532**	**8.9**
Abu Bakr Al-Siddiq	318	26	8.2
Abu Nakhla	208	25	12.0
Airport	241	37	15.4
Al Daayen	148	13	8.8
Al Jumailiya	30	3	10.0
Al Kaaban	52	4	7.7
Al Karaana	67	4	6.0
Al Khor	156	17	10.9
Al Rayyan	272	23	8.5
Al Ruwais	135	4	3.0
Al Sheehaniya	193	11	5.7
Al Thumama	249	22	8.8
Al Waab	209	20	9.6
Al Wajbah	287	28	9.8
Al Wakra	291	11	3.8
Staff Clinic	1,275	102	8.0
Leabaib	349	23	6.6
Leghwairiya	40	7	17.5
Madinat Khalifa	262	22	8.4
Mesaimeer	321	39	12.1
Omar Bin Al Khatab	249	23	9.2
Qatar University	223	22	9.9
Umm Ghuwailina	170	21	12.4
West Bay	256	25	9.8

Despite the increased patient footfall and risk environment, the COVID HCs had an attack rate of 10.1%, which is not significantly different from the average attack rate of 8.9% among staff located in other HCs (*p* = 0.26).

## Discussion

PHCC has taken precautionary measures to prevent the spread of COVID-19 amongst its HCWs. They have maintained vital services such as well-baby and vaccinations, ultrasound, and premarital testing clinics, all by encouraging patients to visit health centers only if medical consultation is imperative. Online health services, through virtual consultations, were provided by PHCC to minimize the risk of exposure and contamination for both patients and medical staff (13).

Designated assessment and triage COVID-19 centers, although having swabbed almost six times more suspected patients per HC than other PHCC HCs, have seen almost similar attack rates amongst their staff when comparing to other PHCC HCs. The similarities in the frequency of infected staff, despite the vast difference in the levels of exposure, can be attributed to continuous training and raising awareness amongst staff on the proper use of PPE and the implementation of stringent infection prevention and control policies and procedures which helps prevent the spread of COVID-19 virus (14).

PHCC has provided adequate education and training content, which includes the use of PPE, hand hygiene, medical waste management, sterilization of patient-care devices, and management of occupational exposure. Within these health centers, non-clinical staff who are predominantly outsourced employees seem to have a higher test positivity and attack rates than the clinical staff.

The higher positivity and attack rates amongst non-clinical staff could be due to several educational, social, and environmental factors such as lack of awareness and training on how to use PPE, less enforcement of occupational safety measures, and crowded accommodations, which is considered to be one of the strong forecasters and substantial contributing risk factors for health problems amongst workers (15). Craft and Manual Workers are more likely to live in crowded shared accommodation in constant proximity to one another, increasing the likelihood of COVID-19 spread through community transmission. They also often gather for social and recreational activities, shared dining, and use of shared equipment (16). The lower positivity among clinical staff can be attributed to the stringent enforcement of infection prevention and control measures, despite the front line aspect of their daily work routine (17). The high volume of patients, combined with an increased need for intensive care, forced HCWs to reorganize care delivery models, in order to minimize risk especially those involving otolaryngologic invasive procedures (18, 19).

Some of these measures include continuously wearing masks, frequent handwashing, and constant availability of sanitizers, in addition to the implementation of social distancing strategies. The administrative staff are considered outliers to the non-clinical staff with low attack rate because they undergo similar safety training as clinical staff and are more likely to live in separate accommodation. Among the clinical workers, pharmacists, dentists, nurses, and wellness staff encountered slightly higher positivity rates, which can be attributed to their nature of work as dedicated COVID-19 swabbing staff. Additionally, pharmacists have frequent dealings with storekeepers and postal department drivers to distribute medication for home delivery. The dental team faces a higher risk of infection due to the oral nature of their work. Nurses and wellness staff have daily close encounters with patients and staff alike, being the first line of contact in the triage selection process.

Although female staff at PHCC outnumber their male counterparts, the spread of the COVID-19 virus has been more pronounced amongst males with higher positivity and attack rates. Some occupations such as storekeeper, security, and engineering & maintenance, predominantly occupied by male staff, have seen considerably high rates of infection. Furthermore, male craft and manual workers, as previously mentioned, are more likely to contract the COVID-19 virus due to the nature of their accommodation and their socio-recreational activities. Various studies have also examined the gender dimension of COVID-19 infection and the epidemiological findings reports have found that male individuals represent in general a higher proportion of the infected COVID-19 patients due to biological, social and economic factors between the genders (20).

According to the analyzed data, staff below 45 years of age have seen higher positivity and attack rates. This could mainly be attributed to the fact that most of the non-clinical outsourced staff are below 45 years of age. Additionally, some of the workers above 55 years of age were allowed to work from home and minimize their daily exposure to the virus through a range of teleconsultation services (21).

In evaluating the transmission of COVID-19 among hospital staff, it is crucial to test both clinical and non-clinical staff during the pandemic to frame the extent of viral spread. Even with limited infection control measures in non-clinical areas, COVID-19 virus transmission did not occur among hospital staff beyond community outbreak, reflecting the effectiveness of infection control measures and appropriate usage of personal protective equipment (22, 23). This also highlights the need to implement the same stringent control measures on non-clinical staff as well, namely outsourced workers, who should undergo training on how to avoid the spread of the virus by taking proper precautionary measures and making appropriate use of their protective equipment. Improvements in their living conditions will ultimately reduce the risk of infection by promoting social distancing and minimizing community transmissions.

These findings highlight the importance of developing a clear and concise national occupational health policy underscoring the importance of training and infection control measures and outlining minimum requirements of health promotion and living environment of staff working in a healthcare setting.

## Resource Identification Initiative

(Cerner Millennium, RRID:SCR_013581)

## Data Availability Statement

The raw data supporting the conclusions of this article will be made available by the authors, without undue reservation.

## Author Contributions

MA-K conceived the study and oversaw overall direction and planning. JA and MK extracted and analyzed the data. MA-K, MA, and AA-N analyzed and interpreted the data. MA-K, SS, and HA-R suggested the different points for the discussion section. MA-K, AA-N, JA, and SS were major contributors in writing the manuscript. All authors discussed the results and contributed to the final manuscript.

## Conflict of Interest

The authors declare that the research was conducted in the absence of any commercial or financial relationships that could be construed as a potential conflict of interest.
